# How cannabis consumption relates to negative affect and expectancies: A cross-sectional survey of an international Spanish-speaking sample

**DOI:** 10.21203/rs.3.rs-6075879/v1

**Published:** 2025-04-02

**Authors:** Luiza Rosa, Jonathon K. Lisano, Renée Martin-Willett, L. Cinnamon Bidwell

## Abstract

Background Research suggests that individuals who use cannabis often have more positive expectations about its effects compared to those who do not. Cannabis use is also associated with increased negative affect, but the impact of these affective symptoms on the relationship between expectation of effects and frequency of use has not been fully explored. Preliminary descriptive outcomes for this study looked at differences in negative affect and expectancies based on cannabis use status, location of residence, and gender. The study’s primary aim investigates if negative affect moderates the relationship between expectations and cannabis use. Methods Participants (N = 421) were recruited nationally and internationally through online platforms and in the community, with an average age of 30 years. Of the sample, 49.4% were female, 53.2% were US-residents, and 47.7% were currently using cannabis. Self-report questionnaires assessed negative affect in the last week, expectations of cannabis effects, and frequency of use. Results Those who use cannabis had significantly higher positive and lower negative expectations compared to those who do not. U.S. residents reported higher positive expectations and lower negative affect than international residents. Regression models showed that positive expectations predicted increased cannabis use, and negative expectations predicted decreased use. A significant interaction was found between positive expectations and negative affect, with negative affect amplifying the positive association between positive expectations and use frequency. Conclusions These results support the role of expectancies on cannabis use patterns and extend the literature to suggest that higher negative affective symptoms strengthen the association between positive expectations and increased use. These findings highlight the importance of addressing both expectations and affect states in a variety of populations when understanding cannabis use patterns.

## Introduction

Globally, it is estimated that 209 million people have used cannabis within the past year (United Nations, 2022). As legalization continues to expand, it is imperative to better understand cannabis' effects on psychological well-being, and how positive or negative perceptions of cannabis influence use. Research suggests positive expectancies may contribute to increased cannabis use (Gali et al., 2021), thus it is particularly important to examine this relationship's link to negative affect and problematic cannabis use. Acute cannabis use has been associated with short-term relief of negative affect (Bidwell et al., 2024). These acute benefits may reinforce use patterns and contribute to risk for heavier use long-term in individuals with affective symptomatology. This relationship may be further confounded by stress, a key component of negative affect, with previous research pointing to the use of cannabis as a coping mechanism in response to stress and stress motivating cannabis use (Hyman & Sinha, 2009; Spradlin & Cuttler, 2019; Moitra et al., 2015). While this evidence suggests that individuals are turning to cannabis to cope with negative affect and stress, more research is needed exploring these effects in diverse cultural settings and how positive or negative cannabis expectancies about cannabis moderate this relationship.

Positive expectancies associated with cannabis may present as believing use reduces stress or anxiety, enhances social interactions, or contributes to creativity (Torrealday et al., 2008). A bidirectional relationship has been observed between positive expectancies and cannabis use, where individuals who use cannabis often hold more favorable beliefs about its effects, which, in turn, drive increased consumption (Elsaid et al., 2024; Buckner, 2013; Martin-Willett et al., 2022). This heightened use further strengthens their positive expectancies, creating a reinforcing cycle. This can in part be explained by the expectancy theory, which posits that individuals are motivated to engage in behaviors they believe will lead to desired outcomes (Lokman et al., 2022). When it comes to negative expectancies associated with cannabis like cognitive impairment, anxiogenic effects, paranoia, and cravings, research varies based on context and individual differences. One study observed that individuals with higher negative expectancies reported more cannabis-related issues (Dekker et al. 2009). Though causality cannot be assumed, this may indicate that these beliefs have a significant impact on the experience and behavior of people who use cannabis.

While these associations between cannabis use and expectancies are well-documented, they may be further influenced by individual differences in negative affective states. For instance, individuals with higher levels of negative affect may be more prone to using cannabis as a coping mechanism if they believe (or have the expectancy) that cannabis would help to improve their negative state. Despite this, research has yet to fully explore how current symptoms of negative affect moderates the relationship between cannabis use and positive and negative cannabis expectancies, particularly across different gender or cultural backgrounds.

Compared to males, females appear to be more convinced of the disadvantages and advantages of cannabis use (Torrejón-Guirado, 2024). However, among those who use cannabis, males are more likely to engage in more frequent use (Cuttler et al., 2016). Research also suggests that pharmacologically, females may be more sensitive to cannabinoids like delta-9-tetrahydrocannabinol (THC) compared to males (Fogel et al., 2017). Current research is also limited by geographical location. Due to varying legal policies, most studies focus on analyzing data from a single country, resulting in a narrow understanding of global perspectives. A recent study, that included both residents of the United States (U.S.) and the Netherlands, found that participants who used cannabis self-reported greater positive and less negative perceptions of cannabis compared to those who did not use cannabis. Further, this effect was significantly greater in U.S. residents compared to their international counterparts (Kroon et al. 2024). Kroon and colleagues emphasized the need for more diverse, cross-cultural research to better understand how cultural and gender differences can significantly influence cannabis attitudes and behaviors. To address this gap, the present study focuses on Spanish-speaking individuals, offering valuable insights into how linguistic and cultural backgrounds shape cannabis-related perceptions and behaviors. By including this population, we aim to contribute to a more diverse and global perspective, as research has traditionally centered around English-speaking populations.

Given the relationships between affect, expectancies, and cannabis use, and a lack of research on gender and cultural differences, this study had a set of three preliminary descriptive goals and one primary aim.  Data were collected via survey responses from an international sample of Spanish-speaking participants and included those who use and don't use cannabis. We first sought to describe the sample and characterize differences in negative affect and positive and negative expectancies depending on 1. Cannabis use or no use status, 2. Residing inside or outside of the United States (U.S.), and 3. Participant gender (Male vs. Female). With these preliminary descriptive aims, we predicted 1) those who used cannabis would have greater positive and lower negative expectancies than those who did not; 2) the participants in the U.S. would show stronger positive expectancies and use cannabis more frequently than international participants, similar to what Kroon et al. (2024) found; and 3) based on previous research, we believe that males will use cannabis more frequently but females will have higher positive and lower negative expectations. Following these preliminary descriptive goals, our primary aim was to assess if negative affect moderated the relationship between positive and negative cannabis expectancies and frequency of cannabis use. Based on the literature, we anticipated that higher levels of negative affect and higher positive expectancy effects of cannabis would be associated with more frequent cannabis use compared to those with low levels of negative affect and low positive expectancies.

### Methods

## Procedures

The data for this study are drawn from a project observing differences in motivation (medicinal vs. recreational) for cannabis use and motivational drives in an international sample of 582 participants (Martin-Willett et al., 2022). The parent study was initially created in English in 2017 as a voluntary and anonymous questionnaire designed to evaluate patterns and perceptions of cannabis consumption in terms of health. The questionnaires were later adapted to include a Spanish translation that was broadly accessible to diverse Spanish speakers (Martin-Willett et al., 2022). After the translation, it was made accessible through an online interface linked to digital research platforms such as mTurk or Prolific. It was also deployed in person as part of a community outreach initiative. The study received Institutional Review Board approval from the University of Colorado, Boulder, and followed all guidelines of the Helsinki Declaration of 1975.

## Participants

To be eligible, participants had to be Spanish speakers (native or primary language) and at least 21 years old. Participants recruited via online platforms (mTurk or Prolific) first completed a screening questionnaire and, if eligible, proceeded to complete the survey. Participants who were recruited in-person received a brief overview and provided informed consent before completing the survey on iPad tablets. Participants recruited online received compensation per the platform policy, while in-person participants did not receive any payment.

## Measures

*Demographics.* Participants were asked to provide information on several demographic variables. Gender was assessed with the question, "What is your gender?" with response options including Male, Female, Non-binary, Prefer to self-describe, and Prefer not to say. Residency was determined by asking participants to indicate their location with options for U.S. State, International, and Unknown. Age was collected through a text box for manual entry in response to the question, "How old are you?" Race was assessed with the question, "What race do you consider yourself to be?" allowing participants to select all that apply from the following options: American Indian or Alaska Native, Asian, Black or African American, Native Hawaiian or Other Pacific Islander, White, Hispanic or Latino, and Prefer not to answer.

*Marijuana Consumption Questionnaire (MCQ)* was used to assess current cannabis use or no use status. If they were currently using cannabis, it also evaluated their frequency of cannabis use in general and by product type (e.g., flower, edibles, concentrates, etc.). Non-use of cannabis status was determined if participants reported that they were not currently using or had never used any cannabis products. Responses were provided using options ranging from “never” to “daily”.

*Brief Marijuana Effect Expectancies Questionnaire (MEEQ-B)* was used to measure participants’ expectations about cannabis’ positive and negative effects. The brief version of the scale consisted of 6 items and was validated by Torrealday et al. (2008) based on the original 48-item MEEQ (Schafer & Brown, 1991). The scale contains two separate components with three items each: positive (MEEQ-Bp) and negative (MEEQ-Bn) expectancies. An example of a positive question is “Marijuana helps a person relax and feel less tense (helps you unwind and feel calm),” while an example for negative is “Marijuana makes it harder to think and do things (harder to concentrate or understand; slows you down when you move).” Participants rated the six items using a Likert scale ranging from 1= “Strongly Disagree” to 5 = “Strongly Agree.” The mean was calculated for both components, with higher scores indicating greater expectancies on each. Based on Cronbach’s alpha, the positive component had a high internal validity ($\alpha = 0.72$), while the negative component had a low Cronbach’s alpha ($\alpha = 0.47$). This is common for measures with only 3 items and consistent with previous research using these measures (Torrealday et al., 2008).

*Negative Affect* was measured with 4 questions on affective experiences over the past week, indicating how often participants were bothered by symptoms of anxiety and depression. These symptoms specifically included feeling nervous, anxious, or on edge; not being able to stop or control worrying; feeling down, depressed, or hopeless; and experiencing little interest or pleasure in activities previously enjoyed. Responses were recorded using a Likert scale ranging from "Not at all" to "Very much." The mean response for the 4 items was calculated, with lower numbers signifying less negative affect. This scale had a high internal reliability ($\alpha = 0.90$).

### Analytical Approach

All statistical analyses were conducted in R statistical computing software (version 4.4.2; R Core Team 2024) utilizing the dplyr (Wickham et al., 2023) and emmeans (Lenth, 2025) libraries. Figures were generated utilizing the ggplot2 library (Wickham, 2016).

### Preliminary Descriptive Aim: Affect and Marijuana Expectancy Effects

*Cannabis Use Status.* ANOVA was used to assess if there were differences in negative affect or positive and negative marijuana expectancy effects based on cannabis use or non-use status.

*Residential Status.* ANOVA was used to compare affect, and positive and negative expectancies between U.S. residents and non-residents.

*Gender.* ANOVA was again used to compare scores for affect, positive and negative expectancies between males and females.

### Primary Aim: Regression Models for Frequency of Cannabis Use

Multiple linear regression was used to address the primary research question of whether affective state moderates the relationship between positive and negative marijuana expectancy effects on the frequency of cannabis use. In this model, affect was used as a predictor in addition to positive and negative marijuana expectancy effects, as well as all two- and three-way interactions (affect x MEEQ-Bp, affect x MEEQ-Bn, MEEQ-Bp x MEEQ-Bn, and affect x MEEQ-Bp x MEEQ-Bn). Tests of simple effects were conducted where significant interactions were present. In models where interactions with any of the predictor variables were significant, low categories were calculated at 1 standard deviation (SD) below the mean, average was the mean, and high was 1 SD above the mean.

### Results

*Final Sample.* A total of 582 participants were recruited nationally and internationally through various online platforms and in-person. Due to the importance of country location to the study aims, 85 participants who preferred not to provide location information were excluded from the analysis. A similar approach was applied to gender: 33 participants with missing data were excluded, and due to limited statistical power, the 1 non-binary and 3 "other" gender participants were not included in the analysis. Finally, 39 people did not complete the measures pertaining to this study and were removed from the subsequent data analysis, giving a final sample of 421 (see Fig. 1).

*Participant Demographics.* The sample for our preliminary descriptive aim consisted of 421 participants, ranging in age from 18 to 70 years ($M$ = 29.71, $SD$ = 9.27). Of these, 49.4% identified as female, and 47.7% reported currently using cannabis products. Most participants identified as Hispanic (50.35%) and White (34.7%; see Table 1 for full demographic details). Regarding residential status, 53.2% resided in the United States, while the remaining participants lived in Europe and South and Central America (see Fig. 2 for specific countries).

Table 1

*Demographic Characteristics of Participants*

| **Sample Characteristics** | ***n*** | **%** |
|---|---|---|
| Gender | | |
| Male | 213 | 50.59 |
| Female | 208 | 49.41 |
| Race/Ethnicity | | |
| Native American | 1 | 0.24 |
| Asian | 8 | 1.90 |
| Black | 7 | 1.66 |
| Hispanic | 212 | 50.36 |
| White | 146 | 34.68 |
| More than 1 Race/Ethnicity | 44 | 10.45 |
| White and Hispanic | 33 | 7.84 |
| Black and Hispanic | 4 | 0.95 |
| Native American and Hispanic | 2 | 0.48 |
| White and Black | 2 | 0.48 |
| Asian, Black, and Hispanic | 1 | 0.24 |
| Asian and Hispanic | 1 | 0.24 |
| Asian and White | 1 | 0.24 |
| Prefer Not To Answer | 3 | 0.71 |
| Residency Status | | |
| United States | 224 | 53.21 |
| International | 197 | 46.79 |
| Cannabis Use Status | | |
| Cannabis Use | 201 | 47.74 |
| No Cannabis Use | 220 | 52.26 |
| *Note. N* = 421 | | |

*Preliminary Descriptive Aim: Group Comparisons of Negative Affect and Marijuana Expectancy Effects.*

Cannabis Use Status: Within the greater sample, participants were grouped based on their use or no use of cannabis within the past year. Individuals who use cannabis had higher MEEQ-Bp ($F(1,412) = 44.80$, $p < 0.001$, *diff* = 0.48) and lower MEEQ-Bn ($F(1,412) = 26.31$, $p < 0.001$, *diff* = -0.37) than those who do not use cannabis. There were no significant differences between cannabis-use and no-use groups regarding negative affect ($F(1,412) = 1.60$, $p = 0.20$). See Fig. 2A.

Residential Status: Participants were classified as U.S. residents or international residents. U.S. residents had significantly higher MEEQ-Bp ($F(1,419) = 4.950$, $p = 0.03$, *diff* = 0.17) and lower negative affect ($F(1,419) = 3.98$, $p = 0.04$, *diff* = -0.21) compared to international residents. No significant difference was observed for MEEQ-Bn based on residential status ($F(1,419) = 2.56$, $p = 0.11$). See Fig. 2B. Among those who use cannabis, it was observed that U.S. residents used cannabis significantly more often over the past month (9.4 ± 10.6 vs. 5.5 ± 8.7 days) than international residents ($F(1,199) = 7.53$, $p < 0.01$, *diff* = 3.94).

Gender: There were no significant differences in MEEQ-Bp ($F(1,419) = 2.05$, $p = 0.15$) or MEEQ-Bn ($F(1,419) = 1.20$, $p = 0.28$) by gender; however, females had significantly higher levels of negative affect ($F(1,419) = 4.18$, $p = 0.04$, *diff* = 0.21). See Fig. 2C. Among the cannabis use group, there were no significant differences in how frequently females or males used cannabis products over the past month ($F(1,199) = 2.66$, $p = 0.10$).

*Primary Aim: Regression Models for Frequency of Cannabis Use.*

Given that cannabis flower is the primary method of consumption (Newport et al., 2023) and this research question aims to address use patterns specifically, we limited data to participants who used cannabis flower at least once per month for this analysis. This provided a sample size of n = 103 for the multiple linear regression (n = 62 participants were excluded for not using cannabis at least once per month, and n = 36 were excluded for not using flower products at least once per month). Results show there were significant main effects of MEEQ-Bp ($B = 4.66$ $SE = 1.62$, $p = 0.005$) and MEEQ-Bn ($B$=-3.21, $SE = 1.59$, $p = 0.04$) on the frequency of cannabis use (Fig. 3). Specifically, increases in MEEQ-Bp were associated with increases in the frequency of cannabis use, and increases in MEEQ-Bn were associated with decreases in the frequency of cannabis use. There was a significant two-way interaction between MEEQ-Bp and negative affect ($B = 4.64$, $SE = 2.17$, $p = 0.03$: Fig. 4) but not MEEQ-Bn and negative affect ($B = 0.69$, $SE = 1.65$, $p = 0.67$). Post-hoc assessment of simple effects for the interaction between MEEQ-Bp and negative affect showed that frequency of cannabis use increased as MEEQ-Bp scores increased in participants with average (*diff* = 5.75, $p = .01$) and high (*diff* = 11.34, $p = 0.001$) levels of negative affect. No other significant interactions were present.

## Discussion

The study's preliminary descriptive analysis was able to provide novel insight in describing how positive and negative expectancies of cannabis differed by cannabis use status, residency, and gender in an international Spanish speaking sample. Further, based on the primary aim of this study, our analysis is the first to describe the moderating effect of negative affect on the relationship between frequency of cannabis use and positive expectancies of cannabis in a Spanish-speaking sample. Our hypothesis for this primary aim was partially supported. There was a significant two-way interaction between positive expectancies and negative affect which observed that participants with average and high negative affect displayed increased frequency of cannabis use as their positive expectancies increased. This was not the case for negative expectancies, as current negative affect and negative expectancies were not associated with the frequency of cannabis use in the past month.

Our descriptive findings relating to use status, found that greater positive and lower negative cannabis expectancies were both associated with more frequent cannabis use, which aligns with prior research (Buckner, 2013; Elsaid et al., 2024). This result reinforces the expectancy theory, which posits that individuals are motivated to engage in behaviors they believe will lead to desired outcomes (Lokman et al., 2022).

Preliminary descriptive outcomes of cultural and gender differences were also explored. U.S. residents exhibited significantly higher positive expectancies and lower negative affect than their international counterparts. This finding is consistent with Kroon and colleagues (2024) and it reflects cultural differences in the perception of cannabis, particularly in regions where cannabis has been legalized and is more widely accepted (Kroon et al., 2024). The difference in both expectancy and negative affect may also relate to different stigma or perceptions of mental health across cultures, suggesting that future research should further investigate how legalization and acceptance of cannabis might influence its use, particularly among populations with higher levels of negative affect. Additionally, while no significant differences in cannabis expectancies or past-month use were observed between genders, females reported significantly higher levels of negative affect compared to males. This finding is consistent with literature suggesting that females may be more susceptible to mood-related disorders (Salk et al., 2017). Understanding these cultural and gender-specific patterns could inform tailored prevention and intervention strategies, especially in regions where cannabis legalization is expanding.

For our primary aim, we found that only positive cannabis expectancies were associated with more frequent cannabis use. This means that individuals using cannabis who are currently experiencing average to high levels of negative affect and simultaneously endorse the positive effects of cannabis use were more likely to have used cannabis over the last month. These findings suggest strong associations between beliefs and use patterns, and that these individuals may be using cannabis to manage their affective states. This is consistent with the literature by demonstrating that positive expectancies are associated with more frequent cannabis use, and extends this relationship by adding current level of negative affect as a moderator of the relationship (Buckner, 2013; Elsaid et al., 2024). Literature supports that the use of cannabis may be effective for reducing negative affect, particularly in cannabis products higher in cannabidiol (CBD; Bidwell et al., 2024). However, Bidwell et al. also points that their research adds to a growing body of literature that reports mixed findings on the associations of cannabis use and negative affect. Some studies suggest that frequent use of cannabis can lead to increased symptoms of negative affect (Troup et al., 2016). While other studies support the notion that moderate use of cannabis products low in THC, the main psychoactive/addictive component of cannabis, and high in CBD may provide acute reductions in negative affective (anxiety) states.

Unlike negative affect and positive expectancies, the present study did not observe a significant interaction among negative affect and negative expectancies on frequency of cannabis use. Previous research on the interaction between negative affect and negative expectancies is limited regarding cannabis use. Similar effects concerning tobacco use have been reported, observing a similar dynamic with negative expectancies not moderating the association between cigarette use and negative affect (Cohen et al. 2002). Cohen et al. also observed that positive reinforcement expectancies moderated the relationship between negative affect and cigarette smoking. These results paired with our present findings suggest that high levels of negative beliefs may not necessarily dissuade individuals from using a substance, especially in the presence of affective symptoms. One potential explanation for this is the presence of coping motivations for use, in which individuals use cannabis despite potential adverse effects because they expect short-term relief from negative affect (Hyman & Sinha, 2009). Additionally, it is possible that the severity of cannabis use disorder plays a role in this relationship. Individuals may continue to use cannabis despite acknowledging its harmful effects, because they have become reliant on it, further reinforcing its use to cope with negative effects (i.e., withdrawal symptoms; Bahji et al., 2020). This is important to consider since negative affect may increase across time as cannabis use increases. Due to the cross-sectional nature of the present study, participants only reported on their current states, which limits understanding of how both use and affect have progressed over time.

### Strengths and Limitations

One of the major strengths of this study is its focus on an international sample and includes only Spanish-speaking participants, a population that has been underrepresented in previous cannabis research. By including individuals from diverse linguistic and cultural backgrounds, this study contributes to a more global understanding of cannabis use behaviors. The inclusion of people who do not use and use regularly also allowed for a more comprehensive analysis of expectancies and negative affect across a broad spectrum of use.

There are limitations to this study that should be considered. This study relied solely on self-report measures, which may introduce response biases. The measure of negative affect, though internally reliable, was limited to only four items assessing anxiety and depression symptoms. A more comprehensive measure with a broader range and including different subcomponents of affect (i.e., depression, anxiety, and stress), may provide a fuller picture of participants' affective states. This would allow future research to assess if the observed relationship reported in this study is reliant on total negative affect or one of its sub-components. Furthermore, the international sample was not fully representative and not evenly distributed across countries, which could limit the generalizability of the findings. Finally, the present study did not analyze differences between THC and CBD use, which has been previously observed to have differing effects on affective state.

## Future Directions

Future research should aim to replicate these findings in more balanced, representative cross-cultural samples, potentially with more granular cultural and geographical distinctions. Additionally, more detailed assessments of negative affect, perhaps using clinical measures, would provide greater insight into how negative affective states influence cannabis use expectancies and behaviors. Finally, the role of cannabis constituents, particularly THC and CBD, should be explored further to determine how these compounds affect the interplay between beliefs and cannabis behavior.

## Abbreviations

CBD, cannabidiol; IRB, Institutional Review Board; MCQ, Marijuana Consumption Questionnaire; MEEQ-B, Marijuana Effect Expectancies Questionnaire – Brief; MEEQ-Bp, Marijuana Effect Expectancies Questionnaire – Brief Positive Subscale; MEEQ-Bn, Marijuana Effect Expectancies Questionnaire – Brief Negative Subscale; THC, Δ9-tetrahydrocannabinol; US, United States.

## Declarations

#### Availability of data and materials

The datasets used and/or analysed during the current study are available from the corresponding author on reasonable request.

#### Competing interests

The authors declare that they have no competing interests.

#### Funding

Funding for the original study was provided by CDPHE2902 (PI: Bidwell) and F31AA029632 (PI: MartinWillett).

#### Authors' contributions

LR took the lead in writing the paper. LR and JL were the primary contributors in conceptualizing and writing the manuscript and analyzing and interpreting the data. RMW designed and funded the study, and substantively revised the manuscript. CB supervised, funded the project, and substantively revised the manuscript. All authors read and approved the final manuscript.

#### Acknowledgements

The authors would like to thank the following individuals for their support: Elicia Azua, Angela Bryan, and Marco Ortiz Torres. The authors also wish to thank the community partners who supported this study, including but not limited to Angela Maria Ortiz Roa, Intercambio, Casa de la Esperanza, the Boulder County Latino Coalition, Sister Carmen Community Center, and Servicios de la Raza.

## Figures

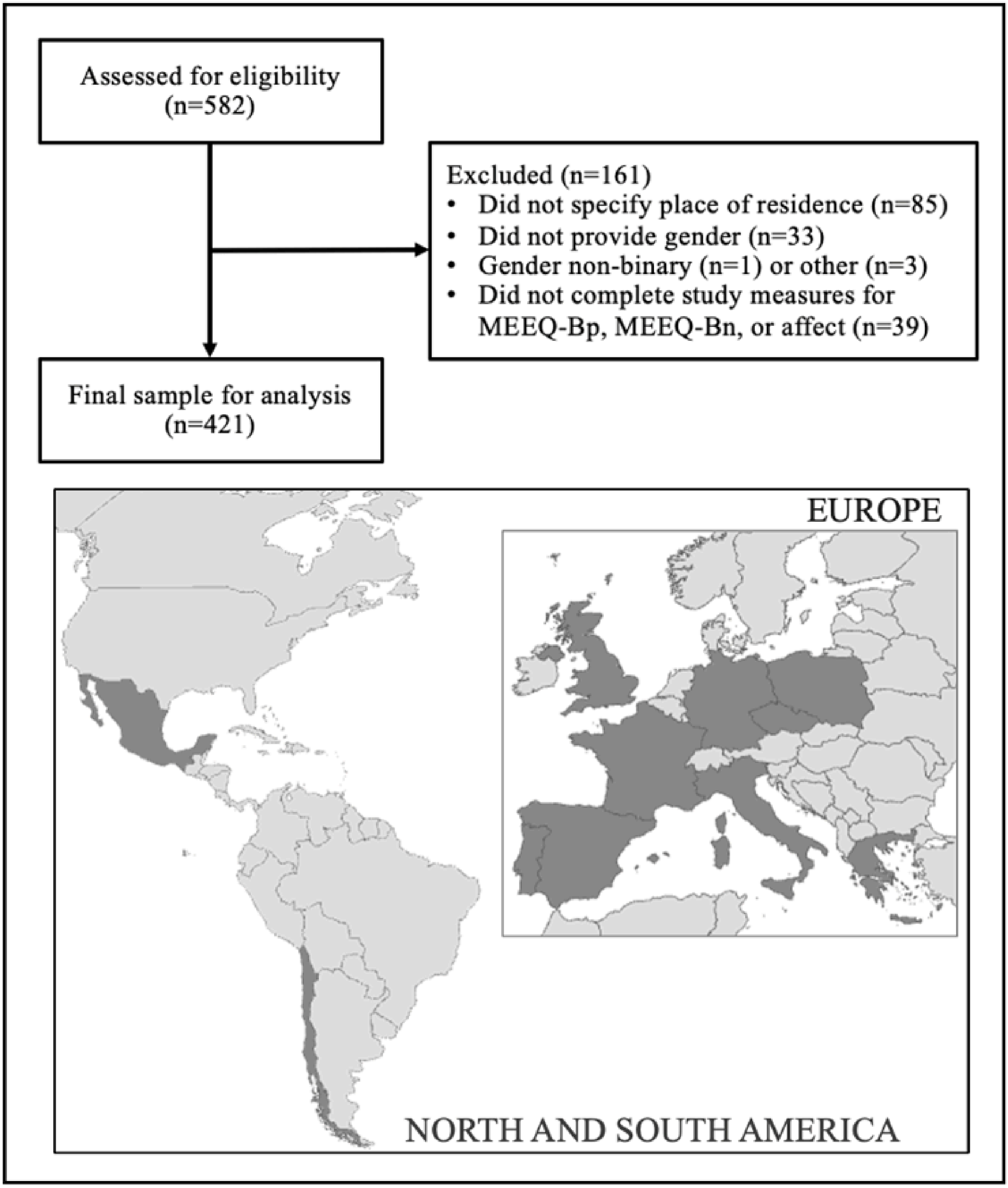

**Figure 1**

*Participant Sample and Countries of residence*

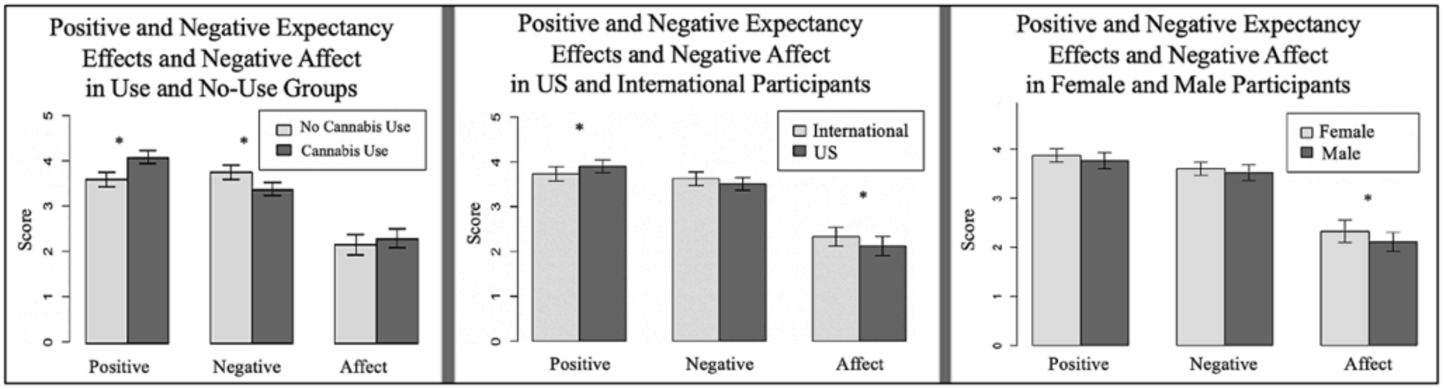

### Figure 2

*Marijuana Expectancy Effects and Affect by Cannabis Use Status, Residency, and Gender*

*Note. Data are the mean ± SE for the Brief Marijuana Effect Expectancies Questionnaire Positive (MEEQ-Bp) and Negative (MEEQ-Bn) subscales and negative affect. A. Score comparisons for MEEQ-Bp, MEEQ-Bn, and negative affect between cannabis use and non-use groups. B. Score comparisons for MEEQ-Bp, MEEQ-Bn, and negative affect between U.S. and international residents. C. Score comparisons for MEEQ-Bp, MEEQ-Bn, and negative affect based on gender at birth. *p<0.05.* ***A.*** *Those who use cannabis have more positive and lower negative expectancies than those who don't, no difference was seen in affect.* ***B.*** *International participants had lower positive expectancies and higher negative affect compared to U.S. counterparts, and no difference in negative expectancies.* ***C.****There were no gender differences in expectancies, but females had more negative affect than males.*

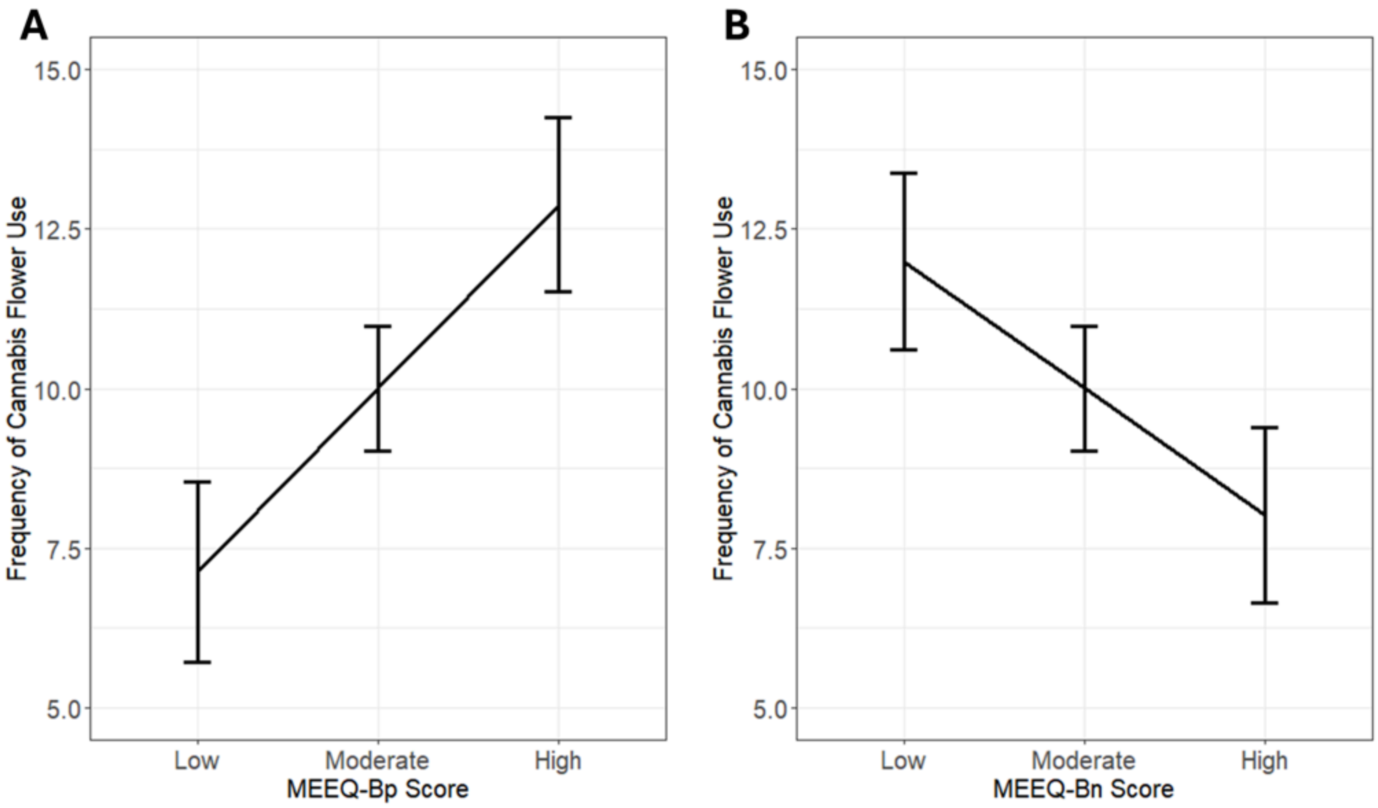

**Figure 3**

*Effects of Positive and Negative Marijuana Expectancy Effects on Frequency of Cannabis Use.*

*Note. Data is limited to participants using cannabis flower at least once per month (N=103). Estimated means ± SE are plotted.* ***A****. Frequency of cannabis flower use by Brief Marijuana Expectancy Effect Questionnaire-Positive Scores (MEEQ-Bp), the more people consumed cannabis, the more positive expectancies they held about it.* ***B****. Frequency of cannabis flower use by Brief Marijuana Expectancy Effect Questionnaire-Negative Scores (MEEQ-Bn), the more people consumed cannabis, the less negative expectancies they held about it.*

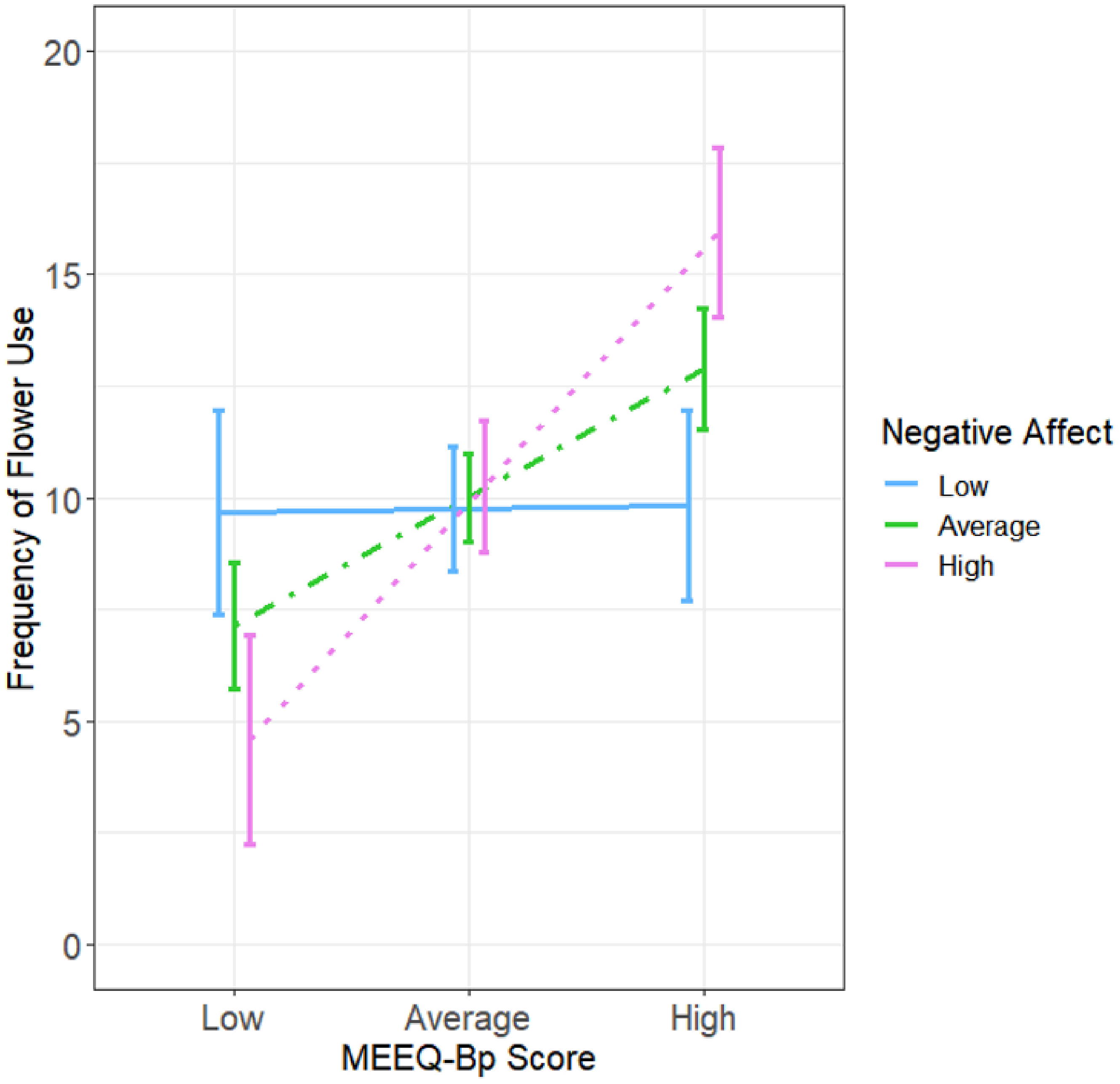

**Figure 4**

*Frequency of Cannabis Use by Positive Marijuana Expectancy Effects and Negative Affect*

*Note. Data is limited to participants using cannabis flower at least once per month (N=103). Estimated means ± SE are plotted. P-values of simple effects are represented by the following: dot-dash p=0.01, dotted p=0.001. Brief Marijuana Expectancy Effect Questionnaire-Positive = MEEQ-Bp. This figure shows a moderation effect, the more people had positive expectancies around cannabis and the more negative affect they experienced in the last 7 days, the more frequently they consumed cannabis.*
